# The Weight of Great Men

**Published:** 1883-11

**Authors:** 


### THE WEIGHT OF GREAT MEN.

A curious letter is just brought to light by the Bangor *Whig and Courier*, in which is recorded the weight of certain Revolutionary officers who were together at West Point a hundred years ago. The letter was written by Joseph May to General David Cobb, of Revolutionary fame, and is as follows:

BOSTON, August 11, 1820.

*Hon. David Cobb, Gouldsborough.*

My Dear General: Your letter of 28th March, written when confined to your house by indisposition, made me, for a moment, feel unhappy—'twas painful—but I have too much respect for you to indulge weak tears when I see you passing the allotted limit of human life; and tho' you find some "labor and sorrow," and tho' "the flocks and the herds afford less pleasure than formerly," yet you rejoice at the vernal sun, you are cheered by the voice of friendship, and when not exercised by actual pain your book affords high employment and enjoyment, which the stranger intermeddles not with. We are marching to a better country, my dear General, where, after a well-spent life, we may hope again to associate with the wise and good whom we have known here—where and how it is to be I am not anxious to know, certain of this, it will be the fittest and best that infinite wisdom and infinite goodness can provide.

Our friend Hayes has lately visited us. He spoke of you repeatedly as a man whom he loved and respected. Looking together over some papers in General Jackson's pocketbook, we found a curious paper, of which I give you a copy:

Weighed at the scales at Westpoint, 19 August, 1788:

General Washington—209 pounds.
General Lincoln—224 pounds.
General Knox—280 pounds.
General Huntington—132 pounds.
General Greaton—166 pounds.
Colonel Swift—219 pounds.
Colonel M. Jackson—252 pounds.
Colonel H. Jackson—230 pounds.
Lieutenant-Colonel Huntington—232 pounds.
Lieutenant-Colonel Cobb—186 pounds.
Lieutenant-Colonel Humphries—221 pounds.

I send you a couple of pamphlets which may amuse you.

Yours affectionately, dear General,

J. MAY.

